# Optimizing the Experimental Method for Stomata-Profiling Automation of Soybean Leaves Based on Deep Learning

**DOI:** 10.3390/plants10122714

**Published:** 2021-12-10

**Authors:** Syada Nizer Sultana, Halim Park, Sung Hoon Choi, Hyun Jo, Jong Tae Song, Jeong-Dong Lee, Yang Jae Kang

**Affiliations:** 1 Department of Applied Biosciences, Kyungpook National University, Daegu 41566, Korea; nizer.sultana@gmail.com (S.N.S.); johyun@knu.ac.kr (H.J.); jtsong68@knu.ac.kr (J.T.S.); 2 Division of Bio & Medical Big Data Department (BK4 Program), Gyeongsang National University, Jinju 52828, Korea; gopm0817@gmail.com (H.P.); sungh716@gmail.com (S.H.C.); 3 Department of Integrative Biology, Kyungpook National University, Daegu 41566, Korea; 4 Division of Life Science Department, Gyeongsang National University, Jinju 52828, Korea

**Keywords:** soybean, stomatal image, deep learning, YOLO

## Abstract

Stomatal observation and automatic stomatal detection are useful analyses of stomata for taxonomic, biological, physiological, and eco-physiological studies. We present a new clearing method for improved microscopic imaging of stomata in soybean followed by automated stomatal detection by deep learning. We tested eight clearing agent formulations based upon different ethanol and sodium hypochlorite (NaOCl) concentrations in order to improve the transparency in leaves. An optimal formulation—a 1:1 (*v*/*v*) mixture of 95% ethanol and NaOCl (6–14%)—produced better quality images of soybean stomata. Additionally, we evaluated fixatives and dehydrating agents and selected absolute ethanol for both fixation and dehydration. This is a good substitute for formaldehyde, which is more toxic to handle. Using imaging data from this clearing method, we developed an automatic stomatal detector using deep learning and improved a deep-learning algorithm that automatically analyzes stomata through an object detection model using YOLO. The YOLO deep-learning model successfully recognized stomata with high mAP (~0.99). A web-based interface is provided to apply the model of stomatal detection for any soybean data that makes use of the new clearing protocol.

## 1. Introduction

Approximately 310,000 plant species grow in various regions worldwide, accounting for nearly 80% of the world’s biomass [1,2]. The yield and biomass of crop plants are determined mainly by the photosynthesis rate increase [3]. Most plants’ leaves have stomata associated with photosynthesis and water loss, playing an essential role in surviving the terrestrial environment. The CO_2_ required for photosynthesis is carried by the stomata, and the number of stomata is associated with CO_2_ conversion rate. Studies have reported that stomatal morphology and development are affected by environmental changes and vary in different species [4]. For a plant in an environment where the CO_2_ concentration is high, its leaves’ stomatal development tends to be suppressed to reduce stomatal density per unit area, reducing the CO_2_ inflow required for photosynthesis [5]. The number of stomata in leaves will be a critical factor in the tradeoff between photosynthetic carbon fixation, closely linked to photosynthesis and crop yield [3,6]. Additionally, the water loss from leaves is controlled by the response of stomatal opening and closure, resulting in delaying the desiccation of plant tissues under extreme environmental conditions. Thus, a proxy to estimate photosynthesis fitness will be the variance in the stomatal density of leaves in diverse agricultural environments such as drought [7,8,9], salinity stress [10], heat stress [11], and precipitation changes [12].

Stomatal density can be a promising target for improving leaf photosynthesis in plants, including soybean (*Glycine max* [L.] Merr) [13]. Several techniques have been developed to capture the stomatal impressions and record stomatal density, shape, and size [14]. Two main approaches for preparing microscopic stomatal images are isolating the epidermis from the mesophyll tissue and producing transparent leaf samples (Table S1).

Usually, a microscopic image of the leaf epidermis by peeling or using clearing agents is used to count the number, size, and shape of stomata manually. This process requires experience, and is costly and time-consuming [15,16]. Therefore, an automated technology was required to assess soybean stomatal density with high precision and efficiency. To accelerate the automation, deep-learning strategies have been suggested. As part of the field of machine learning, the deep-learning method is a popular, recent research technique, especially in the field of image processing. To detect and map image information, this technique relies on a deep neural network [17]. Several deep neural networks are specifically designed for detecting the subregions of query images, such as RCNN (regions with convolutional neural network), Faster RCNN, and YOLO (you only look once) [18]. The pretrained parameters that are already trained on the large dataset such as Common Object in Context (COCO, https://cocodataset.org/ (accessed on 30 September 2021)) can be used for other object detection projects based on the custom datasets. This transfer learning technique also enabled training with few shots learning, which only required a small dataset to train enough. As a result, object detection algorithms have expanded their application areas, and they are now used in plant breeding to detect pests and diseases [19,20].

Stomata Counter is a predictive model using neural networks to count stomatal objects in microscopic images [21] using publicly available microscopic image datasets that use different methodologies. Even though several preparation methodologies were covered, their predictive model is not currently generalized to detect stomata from microscopic images generated by other methods. An automatic segmentation strategy was also used to detect and measure stomatal pores [22]. Similarly, the predictive model may overfit the training image styles, which may differ from other microscopic images. It would be challenging to train the generalized model that works perfectly on several microscopic images; rather, it would be possible to train the specific predictive model for sample preparation and the microscope type.

The perishable nature of fresh leaf samples demands a preservation technique while dealing with many samples. Several studies mentioned that using a preservation technique allowed cross-sections to be further used with orchids (*Orchidaceae*) [23] and Arabidopsis leaves [24]. The concentration of clearing agents is a factor for determining the time to become transparent leaves, which varies between a few hours and several days based on plant species [25]. The optimal method and preservation test of clearing agents to observe stomata in soybean leaves had little information to date. Here, we developed a new modified preparation and preservation method for the microscopic observation of stomata in soybean leaves and its specialized object detection model. We optimized leaf clearing for a clear vision of stomata in microscopic images and created microscopic images of soybean stomata. We trained the object detection model using YOLO [26] based on the image dataset.

## 2. Results

### 2.1. Experimental Method Optimization for Clear Stomatal Pictures

**Figure 1:**
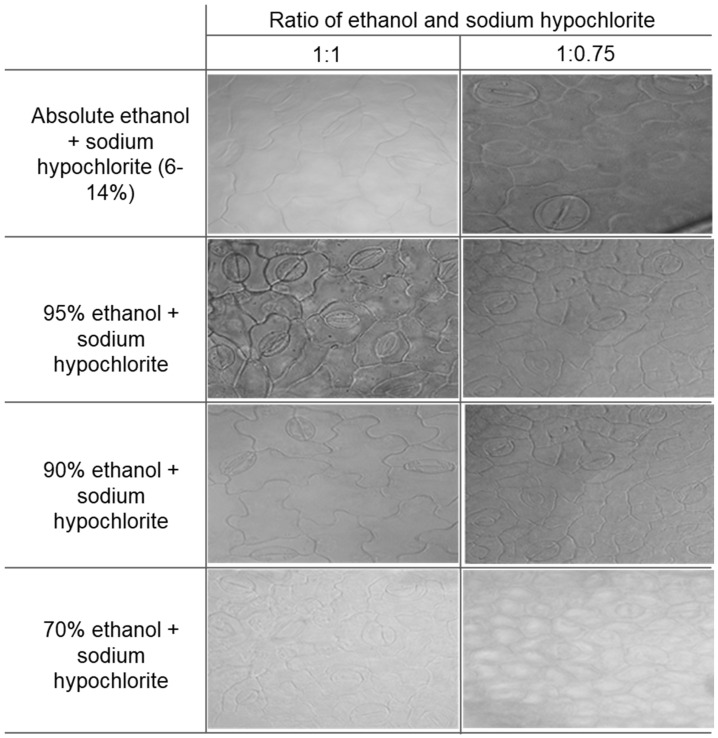
Stomatal microscopic images obtained from 8 different combinations of clearing agents.

To produce high-quality microscopic stomatal images of soybean leaves, we compared eight combinations of clearing agents using soybean leaves (Figure 1). Absolute ethanol and NaOCl in 1:1 (*v*/*v*) and 1:0.75 (*v*/*v*) could make the leaves transparent. However, these clearing agents failed to produce high-quality stomatal images. The mixture of 95% ethanol and NaOCl in 1:1 (*v*/*v*) and 1:0.75 (*v*/*v*) produced transparent leaves with high-quality stomatal images within 1 h. The quality of image was normal for the mixture of 90% ethanol and NaOCl in 1:1 (*v*/*v*), whereas the image quality obtained from the mixture of 90% ethanol and NaOCl in 1:0.75 (*v*/*v*) was low. A mixture of 70% ethanol and NaOCl in 1:1 (*v*/*v*) and 1:0.75 (*v*/*v*) failed to produce a high-quality image. Comparing stomatal images from eight combinations of ethanol and NaOCl, we initially chose a mixture of 95% ethanol and NaOCl in 1:1 (*v*/*v*) and 1:0.75 (*v*/*v*) ratio as a new clearing agent for their excellent performance than other combinations.

**Figure 2:**
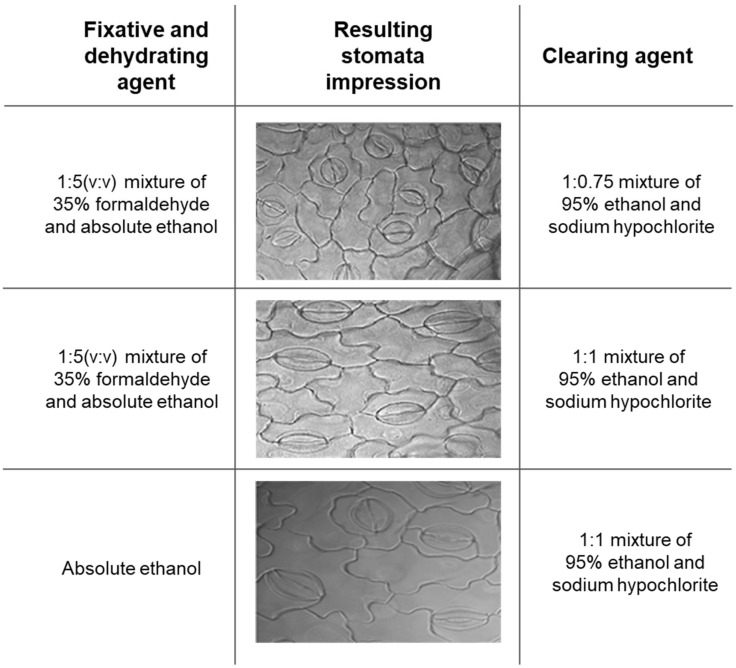
Stomatal microscopic images by a new clearing agent with different fixative and dehydrating agents.

We optimized a new clearing agent in combination with fixative and dehydrative agents, such as three fixative and dehydrating agents with formaldehyde and absolute ethanol (Figure 2). We chose absolute ethanol as fixation and dehydration instead of formaldehyde to compare the three fixative and dehydrating agents. This study has developed a new clearing method for clearing, fixative, and dehydrating agents for their high-quality stomatal image of soybean leaves within 2 h (Figure 2).

**Figure 3:**
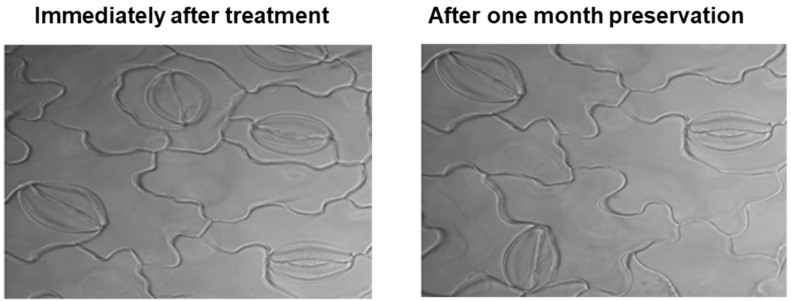
Stomatal microscopic images by preservation test.

To produce large samples for the stomatal images, we conducted a preservation test with a new clearing method. For preservation testing, leaf samples were checked repeatedly. After one month, the stomata’s resolution did not vary in consistency from those assessed immediately after preparing the leaf samples (Figure 3).

**Figure 4:**
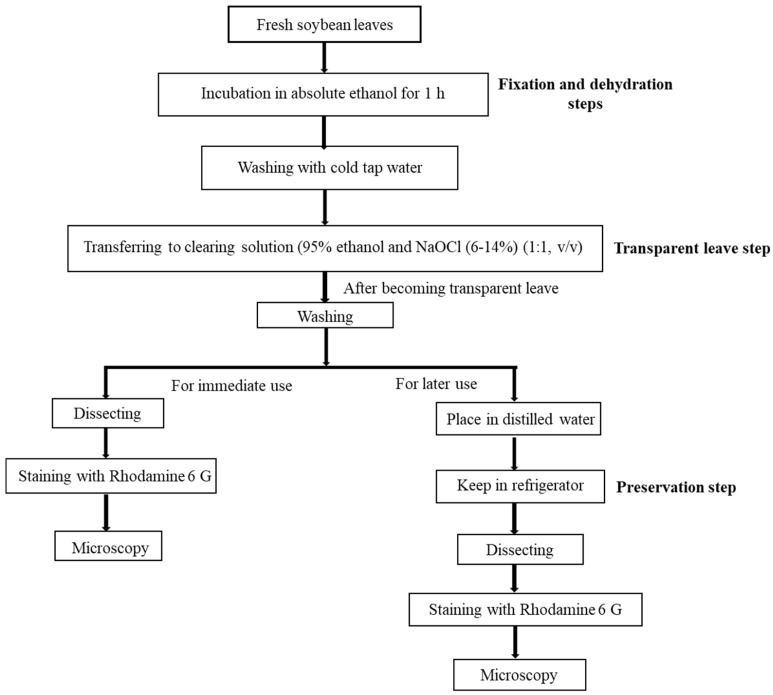
Schematic representation of the workflow of a new clearing method with preservation step.

A flowchart for a new clearing method to prepare the microscopic images for short- and long-time use is presented in Figure 4. Therefore, the new improved method was suggested for the preparation of stomatal images as follows: incubate samples in absolute ethanol for 1 h as fixation and dehydration steps, washing with cold tap water, and transferring samples into a clearing agent (95% ethanol and NaOCl [6–14%] [1:1, *v*/*v*]) for 1 h as the transparency leaves step.

### 2.2. Optimization of Experimental Methods for Automating Stomata-Profiling

**Figure 5:**
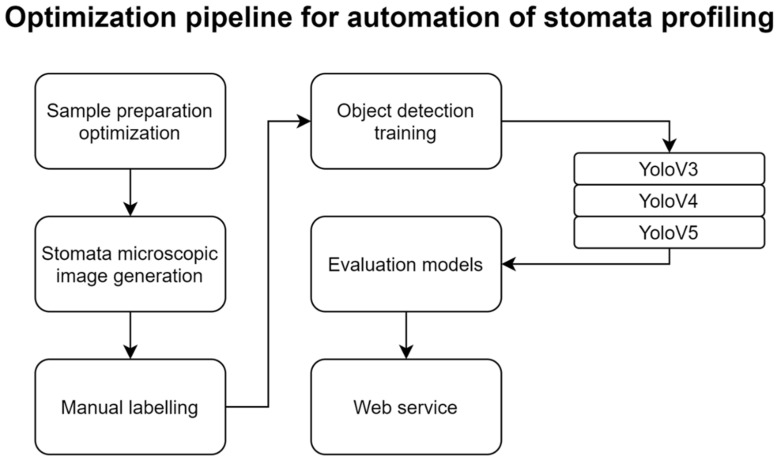
Proposed pipeline optimized for automatic stomata-profiling.

We tested an optimization strategy of experimental methods for the automatic recognition of stomata (Figure 5). It is important to have consistently prepared images that eliminate unnecessary variations by experimental differences for stomata-profiling automation. We attempted to make it simple to replicate the experiments easily. Based on optimized experimental methods, many microscopic images of stomata were continuously obtained and an object detection model was explicitly trained for the experimental method.

#### 2.2.1. Training the Stomata-Detection Model

**Figure 6:**
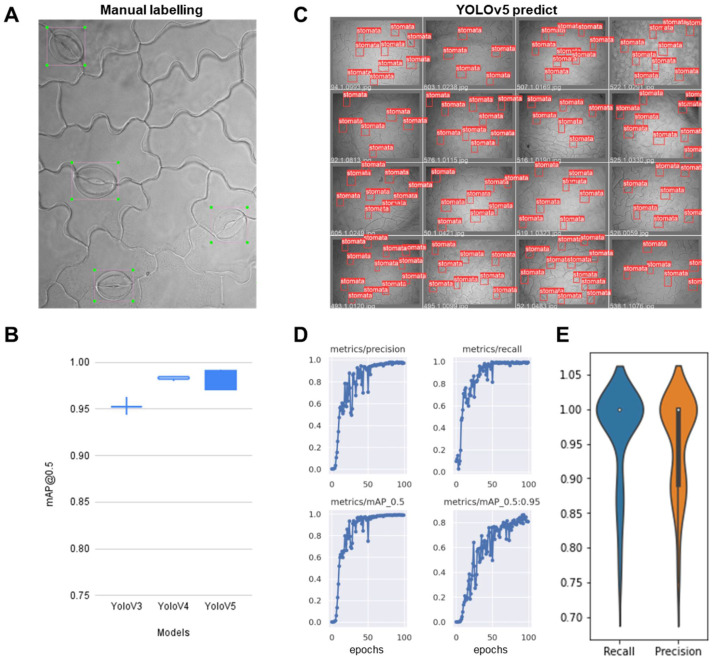
YOLO-based stomatal detection training and its evaluation. (**A**) Preparation of training dataset using manual labeling and the resulting prediction by trained model. (**B**) The statistical evaluation of the trained model by precision and recall metric. (**C**) Stomatal labeled images based on our train YOLO model. (**D**) Model evaluation using validation set during the training based on the metrics including precision (upper left), recall (upper right), mAP_0.5 (bottom left), mAP_0.5:0.95 (bottom right). (**E**) Precision and recall evaluation on a test set with a trained model.

The stomatal objects in 183 images were manually labeled with bounding boxes. Several stomata were blurry because they are microscopic images, but bounding boxes were also annotated on them if human eyes recognize them as stomata (Figure 6A). The images and labels were supplied to the YOLOv3, YOLOv4, YOLOv5 object detection models to retrain the pretrained parameters. We trained each model 10 times with a randomly selected training set to see if the stomata in the test set are well detected, because the number of tagged stomatal images is not very large. Notably, the resulting models showed a very high average mAPs (0.94, 0.98, 0.99) on the testing dataset for YOLOv3, YOLOv4 and YOLOv5, respectively (Figure 6B). This suggests that YOLO models can successfully capture the patterns without very large labeled images, especially for the distinct objects such as leaf stomata (Figure 6C). Especially, recently published YOLOv5 outperformed with regard to evaluation metrics including mAP, precision and recall (Figure 6D). We further evaluated whether stomata’s recognition is correct or not based on recall and precision for each image with additionally labeled 50 stomatal images (Figure 6E). The mean recall and precision were 96.6% and 95.1%, respectively, suggesting that our trained model can recognize the stomata reliably.

#### 2.2.2. Development of Web Applications to Serve the Stomata-Detection Model

**Figure 7:**
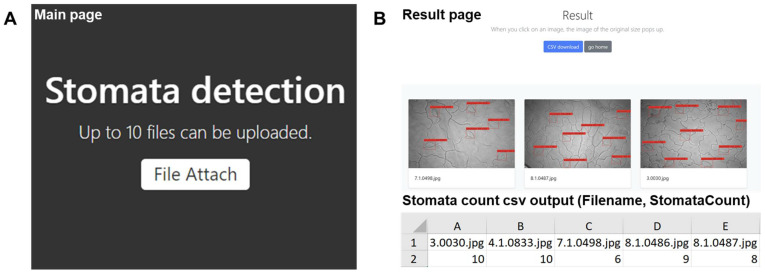
User interface (UI) of web application to serve the trained model. (**A**) Main page. Up to 10 files can be uploaded by the “File Attach” button. (**B**) Result page. The resulting page displays automatically labeled stomatal images and a download button for the stomatal count matrix for each processed image. Red boxes were automatically detected by user interface of web application. The values above the red box represent the intersection over union percentage between a manual bounding box and a predicted bounding box. The count matrix shows the image file names and the number of stomatal counts in CSV file format.

A web page (http://stomata.plantprofile.net/ (accessed on 31 October 2021)) was developed to allow users to easily access and use the stomata-detection model (Figure 7). The web page was created with the Flask web development framework. Our trained YOLOv5 model function is equipped with the Flask and served via Gunicorn (https://gunicorn.org/ (accessed on 30 September 2021)) and Nginx web server [27]. A simple user interface was developed to upload multiple microscopic images to the server. For each file, the server returns the annotated images and stomatal counts to users in Excel format. Presently, we have restricted the number of images that can be uploaded and processed at any one time to 10 image files.

#### 2.2.3. Validation Test Compared Manual Stomatal Counting and Web Applications of Automatic Stomata-Detection Model

To validate the efficiency of stomatal counting by the stomatal detection-model, 386 microscopic images from the datasets were utilized to compare the performance of the trained detection model to manual counting method. There were variations for the number of stomata among soybean accessions (Table S2). Manual counting showed the range from 4.0 to 24.0 with an average value of 9.8 per frame (0.073 mm^−2^). In comparison with manual counting, the automatic counting process revealed the range of stomatal numbers was from 4.0 to 24.0 with a mean value of 9.6. When we converted the stomatal number to stomatal density (per mm^2^), manual counting ranged from 54.8 to 328.8 mm^−2^ with a mean of 134.0 mm^−2^ whereas by automatic detection the range was from 54.79 to 328.8 mm^−2^ with mean of 131.4 mm^−2^. A strong correlation was observed between manual and automatic stomatal counting methods (*r* = 0.98, *p* < 0.0001).

**Figure 8:**
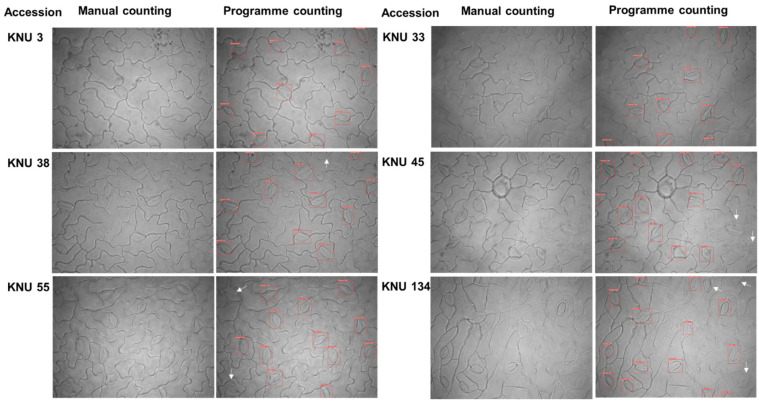
Comparison stomatal detection with six soybean accessions by manual and automatic counting by trained YOLO model. Red boxes were automatically detected by user interface of web application. White arrows were represented not to be detected by trained models.

The stomatal number counting in six accessions by manual and automatic stomatal detection of web application were shown in Figure 8. In the imagery of KNU 3 and KNU 33, all stomata were detected by both manual and automatic stomatal detection by trained models. Automatic stomatal detection has failed to detect two stomata in the imagery of KNU 38, KNU 45 and KNU 55. There were three unrecognized stomata in the imagery of KNU 134. We assumed that stoma were not detected with the low quality of the image. In addition, the parts of stomata at the edge of the image frame were not detected by automatic stomatal detection.

## 3. Discussion

The number of stomata varies from species to species. Cole & Dobrenz [28], reported that the average of stomatal density in alfalfa ranged from 146 to 265 mm^−2^. Stomatal density on the lentil genotypes ranged from 198 ± 25 to 291 ± 36 mm^−2^ [29]. Peksen et al. [30] reported stomatal density of faba beans in lower and upper epidermis of the leaflet ranged from 259.52 to 305.88 mm^−2^ and from 236.66 to 288.26 mm^−2^, respectively. Different studies showed a variation in stomatal density from 242 to 345 mm^−2^ with 43 soybean accessions [31], and from 192 to 334 mm^−2^ with 77 soybean accessions [32], from 93 ± 3 to 166 ± 4 mm^−^^2^ with the 90 soybean accessions [13]. Tanaka et al. [32] showed a significant variation in stomatal density between U.S. cultivars and Japanese soybean cultivars. These results indicate that there is a genetic variation in stomatal density in soybean. In this study, we observed a variation of stomatal density with 386 accessions ranging from 54.79 to 328.8 mm^−2^ with a mean of 134.0 mm^−2^ (Table S2).

The number of stomata in a leaf will be a critical factor in the tradeoff between photosynthetic carbon fixation, closely linked to photosynthesis, response to abiotic stresses [10,11,12] and crop yield [3,6]. Therefore, it is important to understand stomatal density and plant response by various abiotic stresses.

Most of the species have more stomata on the lower (abaxial) surface than on the upper (adaxial) surface of leaves. However, wheat is an exception that stomatal density was higher on the upper surface of leaves than on the lower surface of leaves [33]. Among leguminous crops in faba bean [30], and soybean [32] has the more stomatal density in abaxial than adaxial surface, however, alfalfa [28], pea [34], lentil [29], and peanut [35] had more stomatal density in adaxial surface.

The abaxial side of the leaf samples were used for a new method and automatic detection by web application in this study. We found that a new clearing method can apply for the adaxial side of soybean leaves as well. In the present study, the experiments were conducted using leaf cultivated soybean accessions. Wild soybean is a wild relative of cultivated soybean and has wider genetic diversity than cultivated soybean [36]. Wild soybeans can be an important genetic resource to find favorable genes which improve the ability of soybean cultivar for abiotic stresses. It has relatively small leaf size compared to cultivated soybeans. Through a new clearing method and automatic detection, further research will be required with stomatal density and index in wild soybean.

In this study, the total number of stomata detected from 386 accessions was 3776. However, 98.0% of stomata (3703) was detected by an automatic trained model (Table S2). We assumed that stomata detection failures were due to blurry parts of the image and incomplete stomata at the edge of the image frame. High quality or high resolution of the stomatal image should be obtained during acquisition to overcome these detection failures.

Stomatal size, stomatal area, stomatal perimeter, stomatal spacing, and stomatal index have garnered considerable attention in plant biology research [37,38,39]. Once a way to automatically measure those traits is developed, the efficiency of the research has been improved. For further research, better stomatal image by a new clearing method in this study will be applied for stomatal traits such size, space, and index by deep learning approach. Furthermore, it can be applied to stomatal research with different plant species. Our improved clearing method is expected to be applicable to legume crops and other plant species with minor modifications.

Deep learning is currently a promising technology for developing prediction models and applications. It does, however, suffer from an overfitting problem, as it is nearly impossible to acquire large amounts of data that can cover all-natural scenarios. As a result, using controlled data rather than data that occurs naturally would be advantageous. Through human eyes and our competent YOLO model, our soybean leaf microscopic images were able to show constant numbers of stomata, achieving a mAP of 0.99. This indicates that our object detection model can automatically determine the number of soybean stomata. Following the improved leaf-clearing method and object identification model, this detection model will aid researchers in studying the relationship between stomatal density, total yield, and genetic variables.

In this study, we successfully developed fixation, dehydration, clearing, and preservation methods for generating clear microscopic stomatal images with many soybean samples that would better suit deep learning. This new clearing method is simple and would reliably create microscopic stomatal images that would eliminate unwanted noise.

Peeling methods, such as nail polish and cuticle-preparation methods, are widely used to observe stomatal morphology. The clearing process starts with leaf treatment for decolorization (removing chlorophyll) [40] and fixation (to lock the tissue to its original status) [41]. After fixation, the crucial step is dehydration, followed by using a new clearing agent to make the tissue more transparent than in other studies [42]. Formalin-based fixatives, which are carcinogenic to humans, were used in other studies’ fixation processes [43]. Instead of using formaldehyde for fixation and dehydration, ethanol was used as a fixative and dehydrating agent in our new clearing method [42,44]. The removal of dark pigments that could have been produced during the initial clearing process was performed in combination with ethanol and NaOCl [45]. A new clearing method is a convenient way to clear leaves within a short time, and leaf epidermis was noticed without visual distraction. It can take several days or weeks for leaf clearing using traditional methods [46]. In contrast, this new clearing method could provide a clear view of leaf adaxial (upper) and abaxial (lower) epidermal surfaces 1 h with treatment Moreover, we mentioned the simplest preservation method ever by which image acquisition of many samples will be convenient.

With only 183 manual bounding boxes labeling, the YOLO model was able to efficiently train the detection of soybean leaf stomata despite image blurring. Only the microscopic images from our method would be properly labeled by our trained model, as we aimed for object detection that would fit our controlled experiments. In addition, the result was a well agreed-upon validation test with 386 accessions. A strong correlation was observed between manual and automatic stomatal counting methods (*r* = 0.98, *p* < 0.0001). Even if the experimental setup changes, our stomatal identification model can be quickly retrained using a transfer learning technique with little labeling effort. Stomata-profiling automation based on deep learning will lower phenotyping labor costs and speed up other analyses, such as genome-wide association or quantitative trait loci studies, which require extensive and precise phenotypic data to discover loci regulating agricultural traits.

## 4. Materials and Methods

### 4.1. Soybean Materials

#### 4.1.1. Experiment 1 (Development of a New Leaf-Clearing Process and Preservation Test)

To develop modified preparation and preservation methods for the microscopic observation of stomata in soybean leaves, a soybean cultivar, Cheongja 3 [47], was used. Soybean was planted at the greenhouse of Kyungpook National University (KNU) in Daegu, Korea (35°87′14″ N, 128°60′14″ E) in the winter of 2019. Seeds were planted on a 50-hole tray (530 mm × 270 mm × 110 mm) containing horticultural soil (Hanareum, Shinsung Mineral, Seongnam-si, Korea). Three seeds were planted and thinned to a final stand of one seedling per hole of tray. Three leaves of fully expanded unifoliate and trifoliate were randomly sampled from each hole 30 d after planting. The size of a unifoliate leaf and a trifoliate leaf were approximately 20 cm^2^ and 128 cm^2^, respectively.

#### 4.1.2. Experiment 2 (Model Development for Automated Stomata-Profiling)

To develop an automatic stomatal detector based on deep learning, 386 soybean accessions from the Korean soybean core collection of *Glycine max* from the Rural Development Administration in Jeonju, Korea, were used to produce the microscopic stomatal images. The entire accessions were planted on 6 March 2020, at the greenhouse of KNU [48]. Two seeds from each soybean accession were planted on 50-hole trays containing horticultural soil and thinned to a final stand of one seedling per hole of tray. Three leaves of fully expanded unifoliate and trifoliate were randomly sampled from each accession 30 d after planting.

### 4.2. Modified Clearing Agent Method and Preservation Test to Capture Stomatal Impressions

To determine and produce high-quality microscopic soybean stomatal images using clearing agents and conduct preservation tests for a large number of samples for deep learning, eight combinations of clearing agents based on different ethanol and sodium hypochlorite (NaOCl) concentrations were tested as follows; absolute ethanol:NaOCl (1:1, *v*/*v*), absolute ethanol:NaOCl (1:0.75, *v*/*v*), 95% ethanol:NaOCl (1:1, *v*/*v*), 95% ethanol:NaOCl (1:0.75, *v*/*v*), 90% ethanol:NaOCl (1:1, *v*/*v*), 90% ethanol:NaOCl (1:0.75, *v*/*v*), 70% ethanol:NaOCl (1:1, *v*/*v*), and 70% ethanol:NaOCl (1:0.75, *v*/*v*). These clearing agents were tested to remove dark pigments.

We tested a new clearing agent for transparency leaves combined with fixative and dehydrative agents to evaluate the efficiency of new clearing processes. The combinations were tested as follows: a 1:5 (*v*/*v*) mixture of 35% formaldehyde and absolute ethanol for a fixative with a 1:1 (*v*/*v*) mixture of 95% ethanol and NaOCl, a 1:5 (*v*/*v*) mixture of 35% formaldehyde and absolute ethanol for a fixative with a 1:0.75 (*v*/*v*) mixture of 95% ethanol and NaOCl for clearing, and absolute ethanol for a fixative with a 1:1 (*v*/*v*) mixture of 95% ethanol and NaOCl for clearing. Whole leaf should be submerged in a 30 mL solution during fixation and clearing processes. Leaf sample fixation and dehydration were rapidly conducted at room temperature; the leaves were withdrawn from the solution when they became transparent and were gently washed with tap water. The prepared leaf tissues were stained with 1 μM of Rhodamine 6G [49]. Bright-field microscopy with a Leica DM2500 optical microscope (Leica Microsystems Limited, Balgach, Switzerland) was used to observe the stomata. Images were captured in a 3840 × 2880 format with a DFC 450C-744780815 camera. Grayscale images and high dynamic range quality at 40× magnification were captured using the Leica application suite (LAS v.4.6) in a 0.3118 × 0.233 mm format.

To conduct a preservation test, trifoliate leaves were collected from the cultivated soybean, Cheongja 3, 30 d after planting. After collection, leaf samples were incubated in absolute ethanol, and after successive removal of chlorophyll, leaf samples were transferred to a mixture of 95% ethanol and NaOCl solution in 1:1 (*v*/*v*). After becoming transparent, leaf samples were transferred for washing with cold tap water. Cleared leaf samples were then placed in petri dishes with cover in distilled water and kept in a refrigerator (4 °C) for the preservation test.

### 4.3. Training Object Detection Model

The algorithm for object detection is an automatic technique that distinguishes objects in a specific picture from the context. In this study, pretrained YOLO [35] was chosen to retrain the model using stomatal images. To prepare training image dataset, 183 labeled images were created to predefine the stomata as target objects with the labeling (https://github.com/tzutalin/labelImg (accessed on 30 September 2021)) open-source software to give prior information about the size and shape of the object. Bounding boxes were manually assigned to the stomata and labeled. Even if the stomatal boundary is blurred or the outline is not obvious, all objects that were identified as stomata by human eyes were named, designated as stomata, and saved as files in the XML format. The prepared dataset was supplied to the Keras deep-learning framework to train the YOLO [50]. The predefined parameters were batch size: 16, learning rate: 0.0001, number of epochs: 100, and probability threshold for object detection: 0.3.

## 5. Conclusions

This study presents a complete workflow of stomatal density analysis. In this study, the proposed methodologies combined a stomatal observation method and an automatic stomatal recognition model by deep learning. Among different established stomatal observation methods, a new clearing method produces high-quality images and reduces sample preparation time for many samples. Furthermore, automated stomatal recognition systems could rapidly process stomatal images, removing the need for researchers to measure the number of stomata manually. The simplicity of a new clearing approach will significantly speed up the investigation of stomatal density on a large scale and will be beneficial for investigators in this field.

## Data Availability

The datasets generated during this study are available from the corresponding author on reasonable request. The web application for stomatal detection is available at http://stomata.plantprofile.net/ (accessed on: 31 October 2021).

## Supplementary Materials

The following are available online at https://www.mdpi.com/article/10.3390/plants10122714/s1, Table S1: The representative methods for detecting stomata in different species, Table S2: Comparison stomata density between counting by manual and develped program.
